# Comparative effect of angiotensin II type I receptor blockers and calcium channel blockers on laboratory parameters in hypertensive patients with type 2 diabetes

**DOI:** 10.1186/1475-2840-11-53

**Published:** 2012-05-17

**Authors:** Yayoi Nishida, Yasuo Takahashi, Tomohiro Nakayama, Satoshi Asai

**Affiliations:** 1 Division of Genomic Epidemiology and Clinical Trials, Advanced Medical Research Center, Nihon University School of Medicine, 30-1 Oyaguchi-Kamimachi, Itabashi-ku, Tokyo, 173-8610, Japan; 2 Division of Clinical Trial Management, Advanced Medical Research Center, Nihon University School of Medicine, 30-1 Oyaguchi-Kamimachi, Itabashi-ku, Tokyo, 173-8610, Japan; 3 Division of Pharmacology, Department of Biomedical Sciences, Nihon University School of Medicine, 30-1 Oyaguchi-Kamimachi, Itabashi-ku, Tokyo, 173-8610, Japan; 4 Division of Laboratory Medicine, Department of Pathology and Microbiology, Nihon University School of Medicine, 30-1 Oyaguchi-Kamimachi, Itabashi-ku, Tokyo, 173-8610, Japan

**Keywords:** Angiotensin II receptor blocker (ARB), Calcium channel blocker (CCB), Hematological parameter, Retrospective observational study

## Abstract

**Background:**

Both angiotensin II type I receptor blockers (ARBs) and calcium channel blockers (CCBs) are widely used antihypertensive drugs. Many clinical studies have demonstrated and compared the organ-protection effects and adverse events of these drugs. However, few large-scale studies have focused on the effect of these drugs as monotherapy on laboratory parameters. We evaluated and compared the effects of ARB and CCB monotherapy on clinical laboratory parameters in patients with concomitant hypertension and type 2 diabetes mellitus.

**Methods:**

We used data from the Clinical Data Warehouse of Nihon University School of Medicine obtained between Nov 1, 2004 and July 31, 2011, to identify cohorts of new ARB users (n = 601) and propensity-score matched new CCB users (n = 601), with concomitant mild to moderate hypertension and type 2 diabetes mellitus. We used a multivariate-adjusted regression model to adjust for differences between ARB and CCB users, and compared laboratory parameters including serum levels of triglyceride (TG), total cholesterol (TC), non-fasting blood glucose, hemoglobin A1c (HbA1c), sodium, potassium, creatinine, alanine aminotransferase (ALT), aspartate aminotransferase (AST), gamma-glutamyltransferase (GGT), hemoglobin and hematocrit, and white blood cell (WBC), red blood cell (RBC) and platelet (PLT) counts up to 12 months after the start of ARB or CCB monotherapy.

**Results:**

We found a significant reduction of serum TC, HbA1c, hemoglobin and hematocrit and RBC count and a significant increase of serum potassium in ARB users, and a reduction of serum TC and hemoglobin in CCB users, from the baseline period to the exposure period. The reductions of RBC count, hemoglobin and hematocrit in ARB users were significantly greater than those in CCB users. The increase of serum potassium in ARB users was significantly greater than that in CCB users.

**Conclusions:**

Our study suggested that hematological adverse effects and electrolyte imbalance are greater with ARB monotherapy than with CCB monotherapy.

## Introduction

Angiotensin II type I receptor blockers (ARBs) are well established antihypertensive drugs that are frequently used as the first-line drug for hypertension. Recently, there has been a focus on the beneficial effects of ARBs other than their antihypertensive effect, such as reduction of proteinuria [1] and decreased heart failure risk in patients with chronic heart failure [2]. Calcium channel blockers (CCBs) are also widely used first-line antihypertensive drugs. CCBs are known to decrease the risk of coronary heart disease and non-fatal stroke in patients with hypertension [3], and to decrease proteinuria in patients with chronic renal disease [4]. While ARBs and CCBs have a favorable effect on blood pressure and decrease the risk of several complications, these drugs have some adverse effects. Renin-angiotensin system inhibitors including ARBs are known to cause hyperkalemia [5] and anemia [6,7]. CCBs are known to cause edema [8].

Hypertension and type 2 diabetes mellitus are conditions that frequently coexist [9], both of which carry an increased risk of cardiovascular and renal disease. Hypertension significantly hastens the progression of diabetic nephropathy and increases the risk of cardiovascular events or death in patients with diabetes. On the contrary, lowering blood pressure decreases albuminuria in type 2 diabetes [10,11]. On the other hand, ARBs have a beneficial effect that prevents the new-onset of diabetes [12], and there has been a recent focus on the effect of ARBs on glucose metabolism. We demonstrated a favorable effect of ARB monotherapy on glucose metabolism in non-diabetic hypertensive patients [13]. Whether ARBs have a favorable effect on laboratory parameters, including parameters of glucose metabolism in diabetic hypertensive patients, may be of clinical significance.

Some randomized clinical studies have compared the adverse effects of ARBs and CCBs. [14-16]. However, those studies usually focused on the adverse events of antihypertensive drugs, and there are few large-scale studies focused on the effects of the drugs on laboratory parameters. In addition, few studies have targeted ARB and CCB monotherapy using a clinical database reflecting 'real-world' data. Therefore, in this study, we evaluated and compared the effects of ARB and CCB monotherapy on laboratory parameters, including parameters of lipid metabolism, glucose metabolism, renal function, hepatic function and hematological analysis in patients with concomitant hypertension and type 2 diabetes mellitus, using a clinical database.

## Materials and methods

### Data source

This was a retrospective database study using the Nihon University School of Medicine (NUSM) Clinical Data Warehouse (CDW). NUSM's CDW is a centralized data repository that integrates separate databases, including an order entry database and a laboratory results database, from the hospital information systems at three hospitals affiliated to NUSM. The prescribing data of over 0.5 million patients are linked longitudinally to detailed clinical information such as patient demographics, diagnosis, and laboratory results data. The schema of NUSM's CDW has been reported by Takahashi et al. [17].

### Study population

For this study, we identified type 2 diabetes mellitus patients with mild to moderate hypertension aged over 20 years, who had been newly treated with ARB monotherapy (n = 922) or dihydropyridine CCB monotherapy (n = 731) for at least two months between Nov 1, 2004 and July 31, 2011. The antihypertensive drugs used in the ARB and CCB monotherapy groups are listed in Table 1. We compared new users of ARBs (n = 601) with propensity-score matched samples of new CCB users (n = 601). We excluded patients who had been treated with other antihypertensive drugs (ARB combination drug, angiotensin-converting enzyme inhibitor (ACEI), diuretic, alpha-blocker, beta-blocker, alpha and beta-blocker, alpha-agonist, reserpine, vasodilator, or renin inhibitor) during the study period. The experimental protocol was approved by the Ethical Committee of Nihon University School of Medicine.

**Table 1:** Antihypertensive drugs

**Category**	**Generic name**	**Trade name**	**No. of cases of monothrapy**	
			**Before PS matching**	**After PS matching**
ARBs				
	candesartan cilexetil	Blopress	289	200
	losartan potassium	Nu-lotan	154	87
	olmesartan medoxomil	Olmetec	177	113
	telmisartan	Micardis	141	91
	valsartan	Diovan	161	110
CCBs				
	amlodipine besilate	Norvasc, Amlodin	355	277
	azelnidipine	Calblock	46	38
	benidipine hydrochloride	Coniel	82	66
	cilnidipine	Atelec, Cinalong	41	36
	manidipine hydrochloride	Calslot	25	21
	nicardipine hydrochloride	Perdipine	17	13
	nifedipine	Adalat, Herlat, Sepamit	133	110
	nilvadipine	Nivadil	30	24
	others (barnidipine hydrochloride, efonidipine hydrochloride ethanolate, felodipine, nitrendipine and nisoldipine)	Hypoca, Landel, Munobal, Baylotensin, Baymycard	22	16

### Exposure and measurements

The baseline measurement period (non-exposure period) was defined as within 12 months before the start of ARB or CCB monotherapy. The exposure period (outcome measurement period) was defined as between 2 and 12 months after the start of ARB or CCB monotherapy. The mean exposure of ARB users and CCB users was 243.2 days and 242.1 days, respectively. Laboratory data, including serum levels of triglyceride (TG), total cholesterol (TC), non-fasting blood glucose, hemoglobin A1c (HbA1c), creatinine, sodium, potassium, alanine aminotransferase (ALT), aspartate aminotransferase (AST), and gamma-glutamyltransferase (GGT), hemoglobin, hematocrit, and white blood cell (WBC), red blood cell (RBC) and platelet (PLT) counts, were collected for each individual at the date nearest the start of ARB or CCB monotherapy in the baseline period, and at the date nearest 12 months after the start of ARB or CCB monotherapy in the exposure period.

### Data elements

For each patient, we collected information of patient demographics (age and sex), medical history, use of medication, and laboratory results. Medical history included cerebrovascular disease (ICD-10 code, I60-I69), ischemic heart disease (I20-I25), other heart disease (I30-I52), liver disease (K70-K77), kidney disease (N00-N19), gout (M10), thyroid gland disorder (E00-E07), rheumatoid arthritis (M05-M06), hyperlipidemia (E78.0-E78.5), or proteinuria diagnosed in the 365 days preceding the first date of prescription of ARB or CCB. Drugs used during the 60 days before the start of ARB or CCB monotherapy included hypoglycemic drugs (including insulin and oral hypoglycemic drugs), lipid-lowering drugs (including statins, fibrates and other lipid-lowering drugs), diuretics, immunosuppressive drugs, gout drugs, potassium preparations, antipsychotics, chemotherapeutic drugs, steroids, non-steroidal anti-inflammatory drugs (NSAIDs), proton pump inhibitors, histamine H_2_ receptor blockers and thyroid drugs.

### Statistical analysis

The ARB user group and CCB user group were matched by propensity score using a 5-digit, greedy 1:1 matching algorithm [18-20]. This method is the standard method to reduce bias by balancing covariates between settings, and has been used in many reports. To generate the propensity score, we used covariates including age, sex, medical history (cerebrovascular disease, ischemic heart disease, other heart disease, liver disease, kidney disease, gout, thyroid gland disorder, rheumatoid arthritis, hyperlipidemia and proteinuria) and previous drugs (hypoglycemic drugs including insulin and oral hypoglycemic drugs, lipid-lowering drugs including statins, fibrates and other lipid-lowering drugs, diuretics, immunosuppressive drugs, gout drugs, potassium preparations, antipsychotics, chemotherapeutic drugs, steroids, NSAIDs, proton pump inhibitors, histamine H_2_ receptor blockers and thyroid drugs), as listed in Table 2. We compared the prevalence of all baseline covariates before and after propensity score matching using *t*-test for continuous variables and chi-squared test for categorical data. After propensity score matching, covariance-adjusted and unadjusted generalized linear models (Dunnett-Hsu post-hoc analysis) were fitted to compare the mean values of laboratory parameters at baseline and during the exposure period in ARB users and CCB users, and were used to compare the mean change from the baseline value to the exposure value in ARB users and CCB users. The covariates that were used in the adjusted model included age, sex, medical history and previous medication, as listed in Table 2. All reported P values of less than 0.05 were considered to indicate statistical significance. All statistical analyses were performed with SAS software, version 9.1.3 (SAS Institute Inc., Cary, NC).

**Table 2:** Baseline characteristics before and after propensity score matching

**Characteristics**	**Before matching**			**After macthing**		
	**ARB users (n = 922)**	**CCB users (n = 731)**	**p value**	**ARB users (n = 601)**	**CCB users p value**	**(n = 601)**
Age (mean, SE)	61.7 ± 0.4	66.8 ± 0.35	<.0001 *	65.5 ± 0.4	65.6 ± 0.39	0.8268
Age over 75 years	130 (14.1%)	158 (21.6%)	<.0001 *	110 (18.3%)	113 (18.8%)	0.8238
Women	316 (34.3%)	281 (38.4%)	0.0798	224 (37.3%)	225 (37.4%)	0.9525
Medical history						
Cerebrovascular disease	254 (27.5%)	208 (28.5%)	0.6837	170 (28.3%)	185 (30.8%)	0.3429
Ischemic heart disease	317 (34.4%)	297 (40.6%)	0.009 *	228 (37.9%)	233 (38.8%)	0.7668
Other heart disease	208 (22.6%)	193 (26.4%)	0.0703	156 (26.0%)	149 (34.8%)	0.6427
Gout	28 (3.0%)	43 (5.9%)	0.0046 *	25 (4.2%)	25 (4.2%)	1
Thyroid disorder	266 (28.9%)	155 (21.2%)	0.0004 *	141 (23.5%)	142 (23.6%)	0.9458
Rheumatoid arthritis	85 (9.2%)	68 (9.3%)	0.9538	48 (8.0%)	51 (8.5%)	0.753
Liver disease	481 (52.2%)	404 (55.3%)	0.2098	327 (54.4%)	316 (52.6%)	0.5247
Kidney disease	688 (74.6%)	483 (66.1%)	0.0001 *	407 (67.7%)	411 (68.4%)	0.8046
Hyperlipidemia	860 (93.3%)	678 (92.7%)	0.6764	559 (93.0%)	561 (93.3%)	0.819
Proteinuria	463 (50.2%)	298 (40.8%)	0.0001 *	255 (42.4%)	256 (42.6%)	0.9535
Previous drugs						
Hypoglycemic drugs	226 (24.5%)	188 (25.7%)	0.574	148 (24.6%)	156 (26.0%)	0.5955
Insulin	79 (8.6%)	44 (6.0%)	0.0498 *	36 (6.0%)	42 (7.0%)	0.4823
Oral hypoglycemic drugs	171 (18.5%)	160 (21.9%)	0.0918	126 (21.0%)	129 (21.5%)	0.8324
Lipid-lowering drugs	339 (36.8%)	237 (32.4%)	0.0655	201 (33.4%)	200 (33.3%)	0.9512
Statin	286 (31.0%)	200 (27.4%)	0.1048	171 (28.5%)	169 (28.1%)	0.8981
Fibrate	36 (3.9%)	29 (4.0%)	0.9481	21 (3.5%)	23 (3.8%)	0.7587
Other lipid-lowering drugs	30 (3.3%)	23 (3.1%)	0.902	18 (3.0%)	18 (3.0%)	1
Diuretics	7 (0.8%)	1 (0.1%)	0.0701	1 (0.2%)	1 (0.2%)	1
Immunosuppressive drugs	14 (1.5%)	10 (1.4%)	0.7995	5 (0.8%)	8 (1.3%)	0.4028
Gout drugs	59 (6.4%)	86 (11.8%)	0.0001 *	48 (8.0%)	51 (8.5%)	0.753
Potassium preparations	2 (0.2%)	4 (0.5%)	0.2674	1 (0.2%)	0 (0%)	0.3171
Antipsychotics	38 (4.1%)	19 (2.6%)	0.0921	18 (3.0%)	17 (2.8%)	0.8638
Chemotherapeutic drugs	14 (1.5%)	18 (2.5%)	0.1666	11 (1.8%)	9 (1.5%)	0.652
Steroids	57 (6.2%)	43 (5.9%)	0.7995	32 (5.3%)	33 (5.5%)	0.8985
NSAIDs	284 (30.8%)	236 (32.3%)	0.5193	181 (30.1%)	190 (31.6%)	0.5741
Proton pump inhibitors	126 (13.7%)	82 (11.2%)	0.136	77 (12.8%)	75 (12.5%)	0.8622
H2 blockers	120 (13.0%)	144 (19.7%)	0.0002 *	93 (15.5%)	100 (16.6%)	0.5824
Thyroid drugs	12 (1.3%)	13 (1.8%)	0.4301	10 (1.7%)	9 (1.5%)	0.8171

## Results

Table 2 shows the characteristics of the patients who had been treated with ARB monotherapy or CCB monotherapy, before and after propensity score matching. Before propensity score matching, ARB users were more likely to have thyroid disease, kidney disease, proteinuria and use insulin, and less likely to have ischemic heart disease, gout, use gout drugs and use H2 blockers than CCB users. After propensity score matching, the mean age was 65.5 and 65.6 years, and 37.3% and 37.4% of ARB users and CCB users were women, respectively.

Table 3 shows laboratory parameters at baseline and during the exposure period. In ARB users, the mean values of TC, HbA1c, hematocrit and hemoglobin and RBC count significantly decreased during the exposure period compared with those during the baseline period, after adjustment for age, sex, medical history and previous medication. The adjusted mean value of potassium significantly increased during the exposure period compared with that in the baseline period in ARB users. The adjusted mean values of TG, glucose, creatinine, sodium, ALT, AST, GGT and WBC and PLT counts were not significantly different during the exposure period compared with those in the baseline period in ARB users. In CCB users, the adjusted mean values of TC and hemoglobin significantly decreased during the exposure period compared with those in the baseline period. The adjusted mean values of TG, glucose, HbA1c, sodium, creatinine, potassium, ALT, AST, GGT, hematocrit, and WBC, RBC and PLT counts were not significantly different during the exposure period compared with those in the baseline period in CCB users.

**Table 3:** Unadjusted and adjusted mean (95% CI) laboratory test values according to ARB or CCB use after propensity score matching

**Laboratory test**	**ARBs (n = 601)**						**CCBs (n = 601)**					
	**Unadjusted**			**Adjusted†**			**Unadjusted**			**Adjusted†**		
	**Mean**	**(95%CI)**	**p-value**	**Mean**	**(95%CI)**	**p-value**	**Mean**	**(95%CI)**	**p-value**	**Mean**	**(95%CI)**	**p-value**
TG (mmol/L)												
baseline	1.65	(1.56, 1.73)	0.5113	1.65	(1.56, 1.73)	0.4846	1.63	(1.56, 1.71)	0.2961	1.63	(1.56, 1.7)	0.2604
exposure	1.60	(1.52, 1.69)		1.6	(1.52, 1.69)		1.57	(1.5, 1.65)		1.57	(1.5, 1.65)	
TC (mmol/L)												
baseline	5.20	(5.12, 5.28)	0.0056 *	5.2	(5.13, 5.27)	0.0018 *	5.18	(5.1, 5.26)	0.0351 *	5.18	(5.11, 5.25)	0.0206 *
exposure	5.05	(4.97, 5.12)		5.05	(4.98, 5.12)		5.06	(4.98, 5.14)		5.06	(4.99, 5.13)	
Blood glucose (mmol/L)												
baseline	7.88	(7.63, 8.13)	0.3744	7.88	(7.66, 8.1)	0.3133	7.96	(7.7, 8.23)	0.3809	7.96	(7.72, 8.2)	0.3257
exposure	7.72	(7.47, 7.97)		7.72	(7.5, 7.94)		7.79	(7.53, 8.06)		7.79	(7.55, 8.03)	
HbA1c (%)												
baseline	6.97	(6.86, 7.08)	0.0451 *	6.97	(6.88, 7.05)	0.0074 *	6.93	(6.81, 7.05)	0.2981	6.93	(6.84, 7.02)	0.1793
exposure	6.81	(6.7, 6.92)		6.81	(6.73, 6.89)		6.84	(6.73, 6.96)		6.84	(6.75, 6.93)	
Creatinine (μmol/L)												
baseline	72.8	(70, 75.5)	0.2045	72.8	(70.7, 74.8)	0.0934	73.8	(70, 77.7)	0.5707	73.8	(71.3, 76.4)	0.3887
exposure	75.3	(72.5, 78)		75.3	(73.2, 77.3)		75.4	(71.5, 79.2)		75.4	(72.9, 77.9)	
Sodium (mmol/L)												
baseline	141.4	(141.2, 141.6)	0.1841	141.4	(141.2, 141.6)	0.1725	141.9	(141.7, 142.1)`	0.1137	141.9	(141.7, 142.1)	0.0993
exposure	141.2	(141, 141.4)		141.2	(141, 141.4)		141.7	(141.5, 141.9)		141.7	(141.5, 141.9)	
Potassium (mmol/L)												
baseline	4.39	(4.36, 4.42)	0.0351 *	4.39	(4.36, 4.42)	0.0241 *	4.3	(4.26, 4.33)	0.8344	4.3	(4.26, 4.33)	0.8298
exposure	4.44	(4.41, 4.47)		4.44	(4.41, 4.47)		4.29	(4.26, 4.32)		4.29	(4.26, 4.32)	
ALT (U/L)												
baseline	27.2	(25.5, 28.8)	0.0903	27.2	(25.7, 28.6)	0.0577	28.5	(26.4, 30.7)	0.6781	28.5	(26.6, 30.5)	0.6465
exposure	25.2	(23.5, 26.8)		25.2	(23.7, 26.6)		27.9	(25.8, 30)		27.9	(26, 29.8)	
AST (U/L)												
baseline	27.3	(26, 28.6)	0.3521	27.3	(26.1, 28.4)	0.3041	28.2	(26.4, 30)	0.6099	28.2	(26.6, 29.9)	0.5741
exposure	26.4	(25.1, 27.7)		26.4	(25.2, 27.6)		28.9	(27.1, 30.7)		28.9	(27.2, 30.5)	
GGT (U/L)												
baseline	53.2	(46.8, 59.6)	0.4179	53.2	(47.1, 59.4)	0.3967	56.1	(49.3, 62.8)	0.7434	56.1	(49.7, 62.5)	0.7292
exposure	49.5	(43.1, 55.9)		49.5	(43.4, 55.6)		57.7	(50.9, 64.4)		57.7	(51.3, 64.1)	
WBC (x10^9^/L)												
baseline	6.36	(6.21, 6.51)	0.8579	6.36	(6.22, 6.5)	0.8494	6.47	(6.32, 6.63)	0.6118	6.47	(6.33, 6.62)	0.5847
exposure	6.34	(6.19, 6.49)		6.34	(6.2, 6.48)		6.42	(6.26, 6.57)		6.42	(6.27, 6.56)	
RBC (x10^12^/L)												
baseline	4.36	(4.32, 4.4)	0.0015 *	4.36	(4.33, 4.4)	0.0002 *	4.4	(4.36, 4.45)	0.3324	4.4	(4.36, 4.45)	0.2798
exposure	4.26	(4.22, 4.31)		4.26	(4.23, 4.3)		4.37	(4.33, 4.42)		4.37	(4.33, 4.41)	
PLT (x10^9^/L)												
baseline	221.2	(216.4, 226)	0.8006	221.2	(216.8, 225.6)	0.7845	222.4	(216.8, 227.9)	0.2089	222.4	(217.2, 227.6)	0.1825
exposure	222.1	(217.3, 226.9)		222.1	(217.7, 226.5)		227.4	(221.9, 233)		227.4	(222.2, 232.6)	
Hemoglobin (g/L)												
baseline	138.0	(136.7, 139.4)	0.0024 *	138	(136.9, 139.1)	0.0002 *	138.5	(137.1, 139.8)	0.0727	138.5	(137.4, 139.6)	0.0315 *
exposure	135.1	(133.8, 136.5)		135.1	(134, 136.2)		136.7	(135.4, 138.1)		136.7	(135.6, 137.9)	
Hematocrit (mmol/mol)												
baseline	0.407	(0.404, 0.411)	0.0069 *	0.407	(0.404, 0.411)	0.0012 *	0.409	(0.405, 0.413)	0.285	0.409	(0.406, 0.412)	0.2033
exposure	0.400	(0.396, 0.404)		0.4	(0.397, 0.403)		0.406	(0.402, 0.41)		0.406	(0.403, 0.409)	

Table 4 shows the mean changes in laboratory parameters during the exposure period compared with the baseline period. The change in potassium was significantly greater in ARB users compared with CCB users, and the changes in RBC count, hemoglobin and hematocrit were significantly smaller in ARB users compared with CCB users after adjustment for covariates.

**Table 4:** Unadjusted and adjusted mean changes in laboratory parameters values during0020exposure period from baseline

**Laboratory test**	**Unadjusted**			**Adjusted†**		
	**Mean**	**(95%CI)**	**p-value**	**Mean**	**(95%CI)**	**p-value**
ΔTG (mmol/L)						
CCB	-0.058	(-0.134, 0.017)	0.7509	-0.067	(-0.139, 0.006)	0.5062
ARB	-0.041	(-0.116, 0.035)		-0.032	(-0.105, 0.04)	
ΔTC (mmol/L)						
CCB	-0.119	(-0.182, -0.056)	0.4512	-0.123	(-0.185, -0.061)	0.5664
ARB	-0.153	(-0.217, -0.09)		-0.149	(-0.211, -0.087)	
ΔBlood glucose (mmol/L)						
CCB	-0.17	(-0.439, 0.099)	0.9651	-0.177	(-0.447, 0.093)	0.9085
ARB	-0.161	(-0.431, 0.108)		-0.154	(-0.424, 0.115)	
ΔHbA1c (%)						
CCB	-0.087	(-0.175, 0.002)	0.2669	-0.089	(-0.175, -0.003)	0.2887
ARB	-0.157	(-0.246, -0.069)		-0.155	(-0.241, -0.069)	
ΔCreatinine (μmol/L)						
CCB	1.575	(-0.59, 3.74)	0.5503	1.667	(-0.466, 3.801)	0.6275
ARB	2.508	(0.343, 4.673)		2.416	(0.282, 4.549)	
ΔSodium (mmol/L)						
CCB	-0.24	(-0.462, -0.017)	0.8194	-0.239	(-0.458, -0.019)	0.8275
ARB	-0.203	(-0.425, 0.019)		-0.204	(-0.424, 0.016)	
ΔPotassium (mmol/L)						
CCB	-0.005	(-0.037, 0.027)	0.0173 *	-0.005	(-0.037, 0.027)	0.0182 *
ARB	0.05	(0.018, 0.082)		0.05	(0.018, 0.081)	
ΔALT (U/L)						
CCB	-0.639	(-2.463, 1.185)	0.2966	-0.633	(-2.433, 1.167)	0.2871
ARB	-2.012	(-3.835, -0.188)		-2.018	(-3.818, -0.218)	
ΔAST (U/L)						
CCB	0.667	(-0.859, 2.193)	0.1633	0.664	(-0.847, 2.175)	0.1618
ARB	-0.867	(-2.393, 0.659)		-0.864	(-2.375, 0.647)	
ΔGGT (U/L)						
CCB	1.599	(-4.675, 7.873)	0.238	1.648	(-4.644, 7.939)	0.2319
ARB	-3.74	(-10.015, 2.534)		-3.789	(-10.081, 2.502)	
ΔWBC (x10^9^/L)						
CCB	-0.057	(-0.185, 0.072)	0.6864	-0.048	(-0.176, 0.08)	0.8298
ARB	-0.019	(-0.148, 0.109)		-0.028	(-0.156, 0.099)	
ΔRBC (x10^12^/L)						
CCB	-0.032	(-0.058, -0.006)	0.0005 *	-0.032	(-0.058, -0.006)	0.0004 *
ARB	-0.097	(-0.123, -0.072)		-0.098	(-0.124, -0.072)	
ΔPLT (x10^9^/L)						
CCB	5.03	(1.71, 8.35)	0.0825	5.057	(1.793, 8.321)	0.0743
ARB	0.872	(-2.448, 4.192)		0.845	(-2.419, 4.109)	
ΔHemoglobin (g/L)						
CCB	-1.722	(-2.542, -0.903)	0.0476 *	-1.721	(-2.538, -0.904)	0.047 *
ARB	-2.894	(-3.713, -2.074)		-2.895	(-3.712, -2.078)	
ΔHematocrit (mmol/mol)						
CCB	-0.003	(-0.005, -0.001)	0.0103 *	-0.003	(-0.005, -0.001)	0.0092 *
ARB	-0.007	(-0.01, -0.005)		-0.007	(-0.01, -0.005)	

We further analyzed the data divided by sex, because the standard values of hemoglobin, hematocrit and RBC count differ by sex. Table 5 shows the mean changes in laboratory parameters during the exposure period compared with the baseline period after adjustment for covariates, in subclass analysis. In women, the change in potassium was significantly greater in ARB users than in CCB users, and the changes in hemoglobin, hematocrit and RBC count were significantly smaller in ARB users than in CCB users. In men, the mean change in RBC count was significant smaller in ARB users than in CCB users.

**Table 5:** Adjusted mean changes in laboratory parameters during exposure period from baseline by sex

**Laboratory test**	**Adjusted Women**			**Adjusted Men**		
	**Mean**	**(95%CI)**	**p-value**	**Mean**	**(95%CI)**	**p-value**
ΔTG (mmol/L)						
CCB	-0.076	(-0.178, 0.025)	0.3449	-0.061	(-0.162, 0.039)	0.8484
ARB	-0.007	(-0.108, 0.095)		-0.047	(-0.148, 0.053)	
ΔTC (mmol/L)						
CCB	-0.127	(-0.239, -0.014)	0.2907	-0.117	(-0.19, -0.044)	0.9665
ARB	-0.214	(-0.326, -0.101)		-0.115	(-0.188, -0.042)	
ΔBlood glucose (mmol/L)						
CCB	-0.17	(-0.593, 0.253)	0.6908	-0.152	(-0.505, 0.202)	0.8447
ARB	-0.293	(-0.717, 0.131)		-0.101	(-0.454, 0.252)	
ΔHbA1c (%)						
CCB	-0.075	(-0.216, 0.066)	0.0777	-0.092	(-0.201, 0.016)	0.9247
ARB	-0.257	(-0.398, -0.116)		-0.1	(-0.208, 0.009)	
ΔCreatinine (μmol/L)						
CCB	0.475	(-0.776, 1.726)	0.0592	2.346	(-0.967, 5.658)	0.923
ARB	2.202	(0.948, 3.456)		2.578	(-0.73, 5.886)	
ΔSodium (mmol/L)						
CCB	-0.09	(-0.434, 0.254)	0.1124	-0.279	(-0.567, 0.009)	0.3479
ARB	-0.49	(-0.835, -0.145)		-0.082	(-0.37, 0.206)	
ΔPotassium (mmol/L)						
CCB	-0.015	(-0.067, 0.038)	0.0188 *	0.0002	(-0.041, 0.041)	0.2423
ARB	0.075	(0.023, 0.128)		0.035	(-0.006, 0.076)	
ΔALT (U/L)						
CCB	-0.921	(-3.478, 1.636)	0.1991	-0.31	(-2.767, 2.147)	0.5436
ARB	-3.32	(-5.883, -0.758)		-1.393	(-3.847, 1.06)	
ΔAST (U/L)						
CCB	1.125	(-1.37, 3.621)	0.0796	0.59	(-1.334, 2.514)	0.6034
ARB	-2.43	(-4.931, 0.072)		-0.135	(-2.057, 1.786)	
ΔGGT (U/L)						
CCB	-1.498	(-6.821, 3.824)	0.3965	3.176	(-6.48, 12.832)	0.3908
ARB	-4.794	(-10.129, 0.541)		-2.839	(-12.482, 6.804)	
ΔWBC (x10^9^/L)						
CCB	-0.092	(-0.278, 0.094)	0.3973	-0.02	(-0.191, 0.152)	0.7449
ARB	0.023	(-0.164, 0.209)		-0.06	(-0.232, 0.111)	
ΔRBC (x10^12^/L)						
CCB	-0.025	(-0.061, 0.012)	0.0004 *	-0.032	(-0.067, 0.003)	0.0286 *
ARB	-0.12	(-0.157, -0.083)		-0.088	(-0.123, -0.053)	
ΔPLT(x10^9^/L)						
CCB	4.675	(-0.389, 9.738)	0.2057	4.86	(0.551, 9.17)	0.3242
ARB	-0.008	(-5.083, 5.067)		1.776	(-2.528, 6.08)	
ΔHemoglobin (g/L)						
CCB	-1.286	(-2.417, -0.156)	0.0135 *	-1.811	(-2.931, -0.692)	0.2222
ARB	-3.333	(-4.466, -2.2)		-2.804	(-3.922, -1.686)	
ΔHematocrit (mmol/mol)						
CCB	-0.002	(-0.006, 0.001)	0.0076 *	-0.003	(-0.006, 0.0004)	0.0796
ARB	-0.009	(-0.012, -0.005)		-0.007	(-0.01, -0.004)	

## Discussion

In this study, we evaluated and compared the effects of ARB and CCB monotherapy on biochemical parameters including serum TG, TC, non-fasting blood glucose, HbA1c, sodium, potassium, creatinine, ALT, AST and GGT and hematological parameters including hemoglobin, hematocrit, and WBC, RBC and PLT counts in patients with mild to moderate hypertension and type 2 diabetes mellitus. We found a significant reduction of serum TC, HbA1c, hemoglobin, hematocrit and RBC count in ARB users, and a reduction of serum TC and hemoglobin level in CCB users, from the baseline period to during the exposure period. The reductions of RBC count, hemoglobin and hematocrit in ARB users were significantly greater than those in CCB users. The increase of serum potassium level in ARB users was significantly greater than that in CCB users. These results suggest that hematological adverse effects and electrolyte imbalance are greater with ARB monotherapy than with CCB monotherapy.

It is known that renin-angiotensin system inhibitors, ACEIs and ARBs, occasionally cause anemia, while having protective effects on various organs. Valsartan decreases hematocrit in recipients of kidney transplantation [21]. Losartan decreases hematocrit, hemoglobin and erythrocyte count in recipients of kidney transplantation [6,22]. In animals, candesartan decreases hematocrit, hemoglobin, erythrocyte count, and erythropoietin level in the rat [23]. Confirming these previous reports, our 'real-world' study showed adverse effects of ARB monotherapy on hemoglobin, hematocrit and RBC count.

There are some reports that the use of renin-angiotensin system inhibitors, including ARBs, is associated with hyperkalemia. The serum level of potassium is significantly higher in ARB users than in CCB users after renal transplantation [24]. The relative risk of hyperkalemia was 2-fold higher with dual therapy (ARB plus ACEI) than with monotherapy (ARB or ACEI) [25]. Use of ARBs and ACEIs is associated with a high prevalence of hyperkalemia, and the prevalence of hyperkalemia is significantly higher in ARB users than in ACEI users [5]. Supporting these previous reports of hyperkalemia, our study showed that ARB monotherapy caused electrolyte imbalance with respect to the serum level of potassium. Our study, in combination with previous reports, suggested that regular checks of serum potassium level may be advisable in ARB users.

There are few reports of ARBs affecting hepatic function. In patients with hypertension and abdominal obesity, there was no significant difference in the levels of ALT, AST and GGT between the candesartan group and placebo [26]. There was no significant difference in the levels of ALT and AST from baseline to six months of use of losartan in hypertensive diabetic patients [27]. Supporting these reports, there was no statistically significant difference in the serum levels of ALT and AST between baseline and the exposure period in both ARB users and CCB users in our study. In addition, those changes from baseline to during the exposure period were not significantly different between ARB and CCB users. Therefore, the influence of ARB and CCB monotherapy on hepatic function may be minimal and not of clinical concern.

TC and HbA1c levels in ARB users decreased during the exposure period compared to the baseline period in this study. Some ARBs modulate peroxisome proliferator-activated receptor-γ (PPAR-γ), which regulates lipid metabolism and is associated with insulin resistance [28,29]. There are some reports that telmisartan, which is a strong modulator of PPAR-γ, has a favorable effect on glucose metabolism. Telmisartan significantly improved HOMA-IR in hypertensive patients and also significantly decreased HbA1c in type 2 diabetic patients, especially in those with poor glycemic control [30]. Treatment with telmisartan significantly improved the hyper-insulin response to glucose loading in patients with hypertension and obesity showing insulin resistance [31]. The favorable effect of ARBs on lipid and glucose metabolism that we observed may be caused in part by activation of PPAR-γ. Another reason for the decrease in HbA1c level in ARB users in our study may be the effect of the reduction of hemoglobin level. Sinha et al. suggested that both serum hemoglobin and HbA1c levels are significantly increased in patients with treatment of iron-deficiency anemia [32]. Ford et al. suggested that hemoglobin concentration is positively correlated with the concentration of HbA1c [33]. The effect of ARBs on the HbA1c level that we observed may have been partly influenced by the reduction of hemoglobin level.

There was no statistically significant difference in the level of blood glucose between the baseline and exposure periods in ARB users; however, we have previously reported that ARB monotherapy decreases the level of non-fasting blood glucose during a 6-month exposure period in non-diabetic patients with hypertension [13]. This discrepancy could be explained in part by differences in the duration of treatment or history of diabetes mellitus. It is possible that the glucose-lowering effect of ARB monotherapy could be weaker in patients with diabetes mellitus than in non-diabetic patients. We will evaluate these issues in our next study.

A decrease of TC was also observed in CCB users in our study. Nakamura et al. reported that CCBs decrease TC in patients with CKD [34]. Supporting the previous report, our results revealed a beneficial effect on lipid metabolism in CCB users in patients with hypertension and type 2 diabetes mellitus.

Subclass analysis showed that the reduction of RBC count was significantly greater in ARB users than in CCB users, in both men and women. On the other hand, the mean changes of potassium, hemoglobin and hematocrit in women were significantly different between ARB users and CCB users, but were not significantly different in men (Table 5). The reason for this discrepancy may be as follows. First, the effects of ARBs on hematological parameters are stronger in patients with low hemoglobin and hematocrit than in those with high levels. It is well known that there is a sex difference in hematological parameters; RBC count, hemoglobin and hematocrit are generally lower in women than in men. Second, the effect of ARBs on hemoglobin and hematocrit may reflect their effects on hormones. Testosterone is known to increase hemoglobin and hematocrit [35]. However, the reason for this discrepancy between women and men is still unclear.

Our study has several limitations. First, the retrospective and non-randomized nature of the design involved inherent issues of selection bias and confounding. We used rigorous statistical methods to balance potential confounding variables between ARB and CCB users, including propensity score matching. However, their ability to control for differences was limited to variables that were available or measurable. Second, we compared the effects of ARBs and CCBs in this study. However, the effects of ARBs on lipid and glucose metabolism slightly differ among these drugs [36-38], and further studies are needed to compare the effects of individual drugs. Third, we did not fix the daily dosage in both ARB and CCB users, because the achievement of blood pressure goal requires various doses of an agent across different individuals or even in the same individual in clinical practice. This study was not designed to assess the effects of ARBs and CCBs at each dosage, because it is difficult to determine whether or not pharmacodynamics are dose-dependent in clinical settings. However, the findings of our study, using a sophisticated statistical method in a real-world setting, are reliable and informative for clinicians.

## Conclusions

In this study, we observed greater reductions of hemoglobin, hematocrit and RBC count, and a greater increase of serum potassium level in patients who had received ARB monotherapy compared with CCB monotherapy. We observed significant differences between ARB and CCB users, although the mean values of these parameters remained within normal limits during the baseline and exposure periods. On the other hand, there was no significant difference in parameters of lipid metabolism, glucose metabolism and hepatic function and WBC and PLT counts between ARB and CCB users. Our findings support the clinical evidence that ARB therapy is associated with hematological adverse effects and electrolyte imbalance.

## Abbreviations

ARB = Angiotensin II type I receptor blocker; CCB = Calcium channel blocker; NUSM = Nihon University School of Medicine; CDW = Clinical Data Warehouse; ACEI = Angiotensin-converting enzyme inhibitor; TG = Triglyceride; TC = Total cholesterol; HbA1c = Hemoglobin A1c; ALT = Alanine aminotransferase; AST = Aspartate aminotransferase; GGT = Gamma-glutamyltransferase; WBC = White blood cell; RBC = Red blood cell; PLT = Platelet; NSAID = Non-steroidal anti-inflammatory drug; PPAR-γ = Peroxisome proliferator-activated receptor-γ; PS = Propensity score.

## Competing interests

The authors declare that they have no competing interest.
